# Transcriptome dynamics in developing testes of domestic cats and impact of age on tissue resilience to cryopreservation

**DOI:** 10.1186/s12864-021-08099-8

**Published:** 2021-11-23

**Authors:** Olga Amelkina, Andreia M. da Silva, Alexandre R. Silva, Pierre Comizzoli

**Affiliations:** 1 Smithsonian Conservation Biology Institute, National Zoological Park, Washington, DC USA; 2 Laboratory of Animal Germplasm Conservation, Federal Rural University of Semi-Arid - UFERSA, Mossoró, RN Brazil

**Keywords:** Puberty, Vitrification, Testicular tissue, Domestic cat, RNA-seq, Networks

## Abstract

**Background:**

Fundamental knowledge of cellular and molecular mechanisms in developing testicular tissues is critical to better understand gonadal biology and responses to non-physiological conditions. The objective of our study was to (1) analyze transcriptome dynamics in developing testis of the domestic cat and (2) characterize age effects on the initial response of the tissue to vitrification. Tissues from adult and juvenile cats were processed for histology, DNA integrity, and RNA sequencing analyses before and after vitrification.

**Results:**

Transcriptomic findings enabled to further characterize juvenile period, distinguishing between early and late juvenile tissues. Changes in gene expression and functional pathways were extensive from early to late juvenile to adult development stages. Additionally, tissues from juvenile animals were more resilient to vitrification compared to adult counterparts, with early juvenile sample responding the least to vitrification and late juvenile sample response being closest to adult tissues.

**Conclusions:**

This is the first study reporting comprehensive datasets on transcriptomic dynamic coupled with structural analysis of the cat testis according to the age and exposure to cryopreservation. It provides a comprehensive network of functional terms and pathways that are affected by age in the domestic cat and are either enriched in adult or juvenile testicular tissues.

**Supplementary Information:**

The online version contains supplementary material available at 10.1186/s12864-021-08099-8.

## Background

Mammalian puberty is a progressive process in which testis undergoes dramatic developmental and structural changes, and involves complex hormonal and molecular modulation to accomplish both somatic cell proliferation/maturation and the initiation of spermatogenesis [1]. Large differences exist between species in hormonal control of puberty and onset of spermatogenesis, as well as in regulation of spermatogenesis and steroidogenesis [2, 3]. Recent studies in rodents and humans using single-cell RNA sequencing (scRNA-seq) technology enriched our knowledge on the processes happening in testis between infancy and adulthood, and further emphasized high species-specificity and value of animal models [4, 5].

The domestic cat is an essential model for biomedical research as well as conservation of endangered felids [6, 7]. Cat spermatogenic function reaches maturity at the age of 8 to 10 months, with initial activation and first signs of spermatogenesis at 5 to 6 months [8, 9]. This is fundamentally different from the mouse model (spermatogonia begin to differentiate shortly after birth resulting in a synchronous first wave of spermatogenesis) and closer to humans, where spermatogonia are maintained in an undifferentiated state prior to the initiation of puberty [3].

In addition to extensive morphological studies of domestic cat testicular tissue [10, 11], the expression of specific genes has also been investigated in developing testis [12, 13]. However, we still lack deeper knowledge on molecular processes happening during testis maturation in cats, as well as the understanding of differences between immature (neonatal), maturing (pre-, peri-, pubertal), and fully mature (adult) testicular tissue. A deeper understanding of the molecular events happening in testis from infancy to adulthood would contribute greatly to development of male fertility preservation and fertility control in domestic cats and wild felids. Whole transcriptome of testicular tissue during its maturation has been sequenced in several mammalian species, including pig [14], Mongolian horse [15], domestic yak [16], human [17] and mouse [18]. Further studies using scRNA-seq were also recently performed for developing testis in human and mouse [4, 5]. For the domestic cat, there has been no study so far that looked on the whole transcriptome of testicular tissue at different stages of its maturation. The only existing RNA-seq study on cat testicular tissue used three adult males over 2 years old and focused on comparison with sterile hybrids [19].

Sperm cryopreservation remains the standard approach for preserving male fertility in many species, including humans. However, when sperm banking is not possible, preservation of small fragments of testicular tissue offers an alternate way for fertility preservation [20, 21]. Preservation of testicular tissue can benefit not only prepubertal individuals who do not yet produce mature sperm, but also post-pubertal and adult patients and animals who may be azoospermic at the moment of semen collection [22, 23]. A recent report on the cryopreservation of testicular tissue for patients across several centers showed that the age of individuals ranged from 5 months all the way to 35 years old [24]. It is therefore essential to develop testicular tissue preservation protocols optimized for different age groups.

Recently, our group observed a better recovery of immature testicular tissues compared to adult in domestic cat after preservation protocol using microwave-assisted drying [25]. Different separate studies also show that testicular tissue from immature cats tend to be more resilient to cryopreservation protocols compared to adults [26–28]. However, there is no study comparing age groups side by side. We also lack a deeper understanding of the cellular mechanisms occurring in testicular tissues in response to vitrification and warming. RNA-seq has been used to explore the effect of cryopreservation on mammalian sperm cells in ruminants [29], boars [30, 31] and giant panda [32], but no studies looked at transcriptome changes in whole testicular tissue. There is evidence that the intrinsic response of cells to cryopreservation is different depending upon whether the cells are part of a tissue or whether they are isolated in a cell suspension [21]. Scaling up of cryopreservation from a microscopic cellular level to a macroscopic tissue level will introduce heat and mass transfer phenomena [33]. Additionally, freeze-thaw procedures for tissue products must result in recovery of both cell viability and tissue structure [33]. Thus, getting information on the whole transcriptome changes in the tissue during preservation is essential for understanding the mechanisms of stress response and recovery of cells as a system. For instance, our group recently demonstrated how RNA-seq and resulting functional networks can help to understand the response of ovarian tissue to various stresses caused by preservation [34].

The objective of our study was to (1) analyze transcriptome dynamics in developing testis of the domestic cat and (2) characterize age effects on the initial response of the tissue to vitrification. RNA-seq was performed on adult and juvenile cats (juvenile being defined as the development period after the infant stage but prior to full adult sexual maturity [4]) using fresh and vitrified whole testicular tissues. Because this is the first study to look at the whole transcriptome of the domestic cat developing testis, our study focused mainly on characterization of global changes in transcriptome and functional pathways throughout testis maturation. We included conventional methods of histology and DNA integrity analysis to be able to relate our transcriptomic data to the available knowledge.

## Results

### Summary of the acquired dataset

**Fig. 1:**
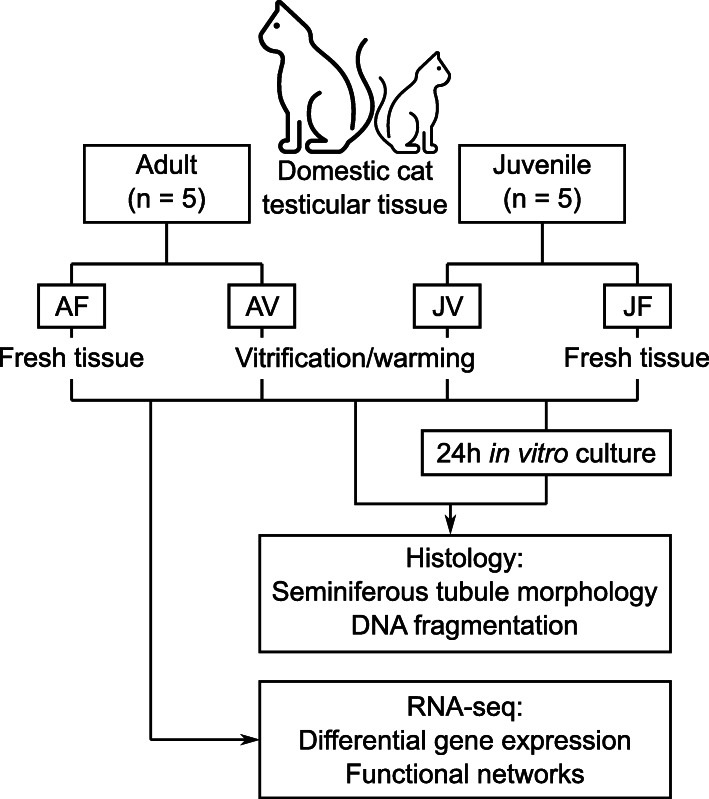
Experimental design

Testicular tissues were obtained from five adult and five juvenile male cats and divided into the following four groups: adult, fresh tissue (AF), adult, vitrified/warmed tissue (AV), juvenile, fresh tissue (JF) and juvenile, vitrified/warmed tissue (JV; Fig. 1). Samples from all four groups were used for histomorphology, TUNEL and RNA-seq analyses. Whole transcriptome of 20 samples was sequenced with one library per each sample, five biological replicates per group, 30 million read depth per sample and 150 bp paired-end read length. The acquired sequence data in fastq format is deposited to NCBI Sequence Read Archive; BioProject accession number is PRJNA741252. Data retrieved after differential gene expression analysis is available in additional files. Data used to create networks is available in the interactive web session view for each network.

### Histology and transcriptome dynamics in fresh testicular tissue from adult and juvenile domestic cats

**Fig. 2:**
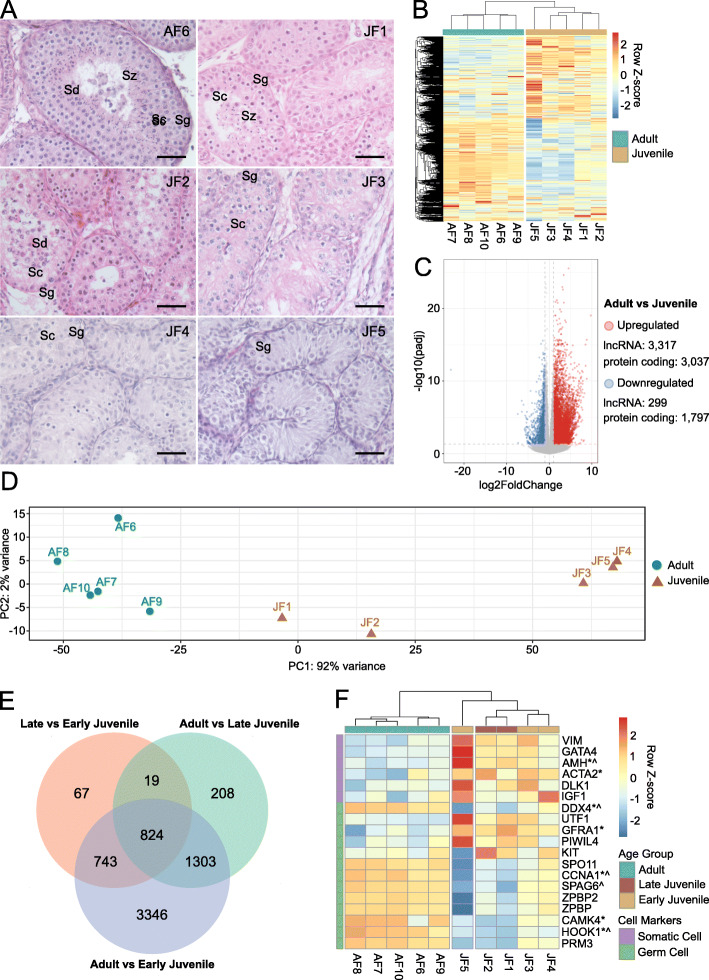
Histology and transcriptome dynamics in fresh testicular tissue of adult and juvenile domestic cats. **A** Histology of testicular tissue samples used for transcriptome analysis; AF6 represents the histology of adult samples. Here and further, sample IDs represent age (A, adult; J, juvenile), tissue condition (F, fresh) and animal (1 to 10). Sg, spermatogonia; Sc, spermatocytes; Sd, round spermatids, Sz, spermatozoa. Scale bar: 50 μm. **B** Heatmap of one-way hierarchical clustering analysis (Euclidean method, complete linkage) using Z-score for RLE normalized values of all genes expressed in testicular tissue from adult and juvenile cats. **C** Volcano plot showing differentially expressed genes in adult vs. juvenile samples (Wald test, adjusted p-value < 0.05, absolute fold change > 2). **D** Principal component analysis plot representing variation in samples from adult and juvenile cats. **E** Venn diagram of differentially expressed genes from 3 comparison pairs: Adult, Late Juvenile (JF1 and JF2) and Early Juvenile (JF3, JF4 and JF5); Wald test, adjusted p-value < 0.05, absolute fold change > 2. **F** Heatmap of one-way hierarchical clustering analysis (Euclidean method, complete linkage) using Z-score for RLE normalized values of selected differentially expressed cell markers. Cell markers have been organized to mark more differentiated germ cell populations as they move from the top to the bottom rows. * - differentially expressed in adult vs. late juvenile; ^ - differentially expressed in late vs. early juvenile

Figure 2 A presents histological overview of samples used for RNA-sEq. All samples from adult cats had Johnsen score above 7, while scores of juvenile samples ranged from 3 to 5 (Table S1, [35]). Unsupervised hierarchical clustering of all genes separated adult and juvenile samples, showing clear transcriptomic division of these age groups (Fig. 2B). Volcano plot in Fig. 2 C represents the results of differential expression (DE) analysis in adult vs. juvenile samples; a total of 8,732 differentially expressed genes (DEGs; Additional file 1). Principle component analysis revealed further division of juvenile samples into seemingly two age groups which we labeled as late (JF1, JF2) and early (JF3, JF4. JF5) juvenile ages (Fig. 2D). DE analysis then was performed using the new three groups with the following comparison pairs: adult vs. late juvenile (Additional file 2), late vs. early juvenile (Additional file 3) and adult vs. early juvenile. Out of all DEGs, 824 were shared between all three comparison pairs (Fig. 2E). All except 2 shared genes had the same expression direction being either continuously upregulated (653 protein coding genes, 140 lncRNAs) or downregulated (11 protein coding genes) from early to late juvenile to adult (Additional file 4). The 19 genes shared only between comparison pairs of late vs. early juvenile and adult vs. late juvenile all had different expression directions (Fig. 2E, Additional file 4).

Heatmap in Fig. 2 F presents clustering of samples based on germ and somatic cell markers (identified from comprehensive data generated in human testicular tissue [4, 36, 37]) that were differentially expressed in adult vs. juvenile. Genes known to be expressed in more differentiated germ cells (SPO11 to PRM3) had higher expression in adult samples when compared to overall juvenile group, while HOOK1, SPAG6 and CCNA1 were also higher expressed in late compared to early juvenile samples showing continuous increase of these markers (Fig. 2 F, Additional file 4). The expression of spermatogonia markers (UTF1 to KIT) was higher in juvenile compared to adult samples with no differences within the overall juvenile group, while the expression of GFRA1 was also higher in late juvenile compared to adult samples (Fig. 2 F, Additional file 2). Somatic cell markers had higher expression in juvenile compared to adult group, with the expression of Sertoli cell marker AMH continuously decreasing from early to late juvenile to adult (Fig. 2 F, Additional file 4). Results from Fig. 2 F in addition with hierarchical clustering based on all genes (Fig. 2B) allowed us to further separate early juvenile group into early I (JF5) and early II (JF3, JF4).

In sum, early I juvenile age group (JF5) presented testis lacking apparent lamina or lumen with only spermatogonia and Sertoli cells present in tubules (Fig. 2 A, Table S1), high expression of undifferentiated spermatogonia marker UTF1, and low expression of differentiating spermatogonia marker KIT (Fig. 2 F). A tubular structure became progressively apparent in early II juvenile (JF3, JF4) with presence of spermatocytes and only few spermatids in some tubules, while a clear defined lamina and lumen were observed across tubules in late juvenile samples (JF1, JF2) with higher number of spermatids present in more tubules (Fig. 2 A, Table S1), as well as significantly higher expression of spermatocyte markers CCNA1 and SPAG6 compared to early juvenile (Fig. 2 F, Additional file 3). Testicular tissue from adult group showed full establishment of spermatogenesis with many spermatozoa present in tubules (Fig. 2 A, Table S1), increased expression of great number of lncRNA compared to juvenile (Fig. 2 C), as well as higher expression of spermatocyte and spermatid markers (SPO11 to PRM3) and lower expression of spermatogonia (UTF1 to KIT) and somatic cell (VIM to IGF1) markers compared to juvenile age group (Fig. 2 F, Additional file 1).

### Changes in functional terms and pathways throughout testicular tissue development in adult and juvenile cats

**Fig. 3:**
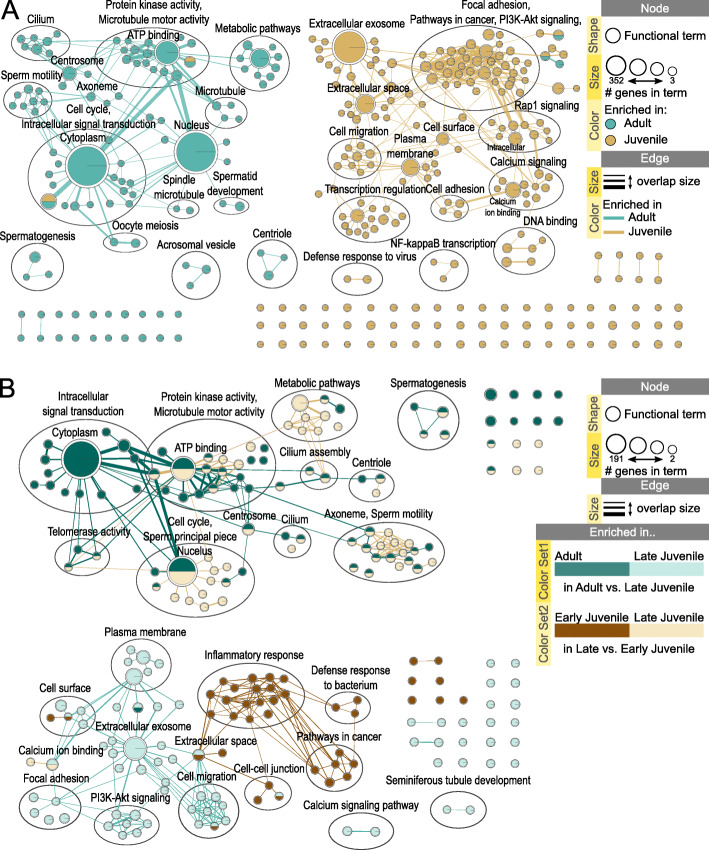
Functional terms and pathways enriched in fresh testicular tissues from different development stages. Enrichment map displays enriched gene-sets based on differentially expressed genes in testicular tissues of (**A**) adult vs. juvenile cats and (**B**) adult vs. late juvenile and late vs. early juvenile cats. Additional file 5 contains the web session of both networks for interactive view

Figure 3 A visualizes DAVID results of functional analysis in an Enrichment map for DEGs from comparison pair adult vs. juvenile. Out of 4,834 protein coding DEGs, 4,550 were annotated in DAVID database for the domestic cat; Gene Ontology and KEGG Pathways databases were used for gene set enrichment. Figure 3B visualizes DAVID results for DEGs from adult vs. late juvenile and late vs. early juvenile comparison pairs. Additional file 5 contains the web session of both networks with interactive view and data table. Functional terms enriched in testicular tissue from adult animals (upregulated in adult compared to juvenile) were mainly related to sperm motility, processes involved in spermatogenesis and fertilization, and cell cycle and division (Fig. 3 A). Majority of these terms were also enriched in adult samples when comparing to late juvenile and in late compared to early juvenile samples, indicating a continuous increase in function of these terms and pathways with testicular development (Fig. 3B). Functional terms enriched in testicular tissues from juvenile cats (downregulated in adult compared to juvenile) were mainly related to cell signaling, cell adhesion, cell migration, as well as terms associated with extracellular region, cell surface, membrane and response to stimulus (Fig. 3 A). Terms related to immune response were specifically enriched in early juvenile samples when comparing with late juvenile (Fig. 3B). Terms related to cell adhesion, membrane and PI3K-Akt signaling were specifically enriched in late juvenile samples when comparing to adult (Fig. 3B).

### Effect of vitrification on histology, DNA integrity, and transcriptome in testicular tissues from adult and juvenile cats

**Fig. 4:**
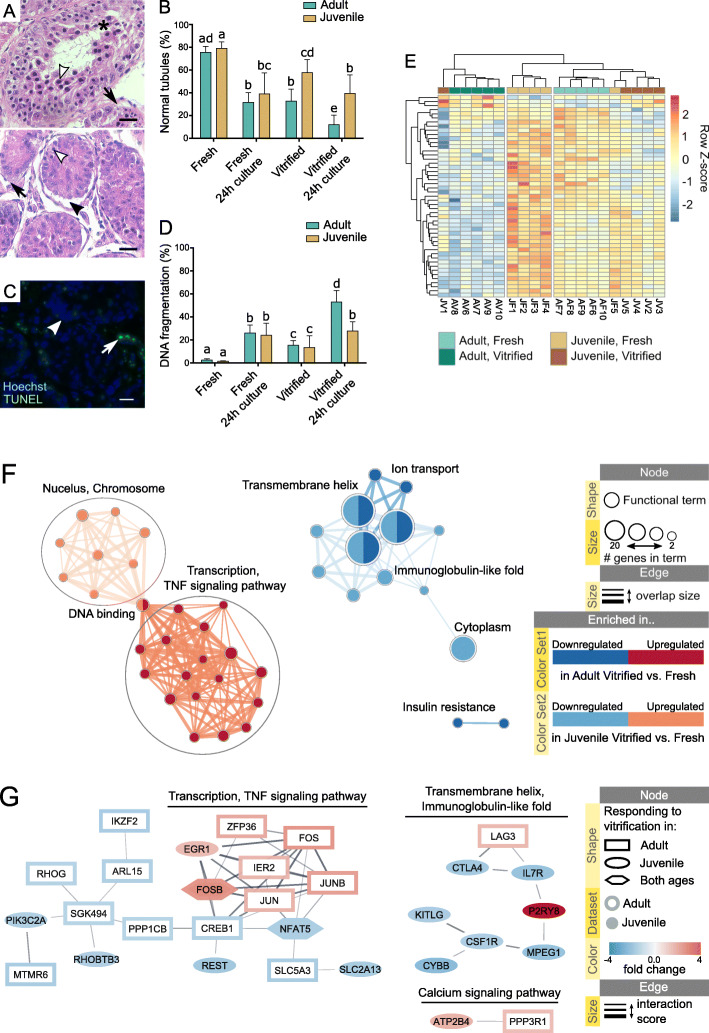
Effect of vitrification on histology, DNA integrity and transcriptomics in testicular tissues from adult and juvenile domestic cats. **A** Representation of morphological damage in vitrified testicular tissue sections showing mature tubule with lumen (upper picture) and immature tubule without lumen (lower picture). Black arrowhead: separation of the basal membrane; white arrowhead: nuclear condensation; black arrows: shrinkage from the basal membrane; asterisk: tubular cell disorganization. Scale bar: 20 μm. **B** Changes in percentage of morphologically normal seminiferous tubules after vitrification and/or 24 h culture. **C** Representation of DNA fragmentation in testicular tissue sections. White arrowhead: intact DNA (Hoechst); white arrow: fragmented DNA (TUNEL). Scale bar: 20 μm. **D** Changes in percentage of DNA fragmentation after vitrification and/or 24 h culture. **E** Heatmap of one-way hierarchical clustering analysis (Euclidean method, complete linkage) using Z-score for RLE normalized values of genes differentially expressed in vitrified vs. fresh samples, independent of age effect (likelihood ratio test, condition effect, adjusted p-value < 0.05). Sample IDs represent age (A, adult; J, juvenile), tissue condition (F, fresh; V, vitrified) and animal (1 to 10). **F** Enrichment map displaying enriched gene-sets based on genes responding to vitrification (p-value < 0.05, absolute fold change > 1.5) in vitrified vs. fresh samples from adult and juvenile cats. **G** STRING network of predicted protein interactions for genes responding to vitrification (p-value < 0.05, absolute fold change > 1.5) in vitrified vs. fresh samples from adult and juvenile cats. Protein interaction clusters were analyzed for functional enrichment within STRING app; clusters with no enrichment were removed from visualization. Additional file 7 contains the web session of both Enrichment map and STRING networks for interactive view

Main types of morphological damage caused by vitrification were cellular disorganization, shrinkage from the basal membrane, and nuclear condensation (Fig. 4 A). The percentage of damaged seminiferous tubules was higher in tissues from adult compared to juvenile cats (Fig. 4B). After 24 h of in vitro culture, warmed tissues from juvenile cats retained a higher percentage of morphologically normal seminiferous tubules compared to adult (Fig. 4B). DNA damage could not be attributed to specific cell types and was observed in different parts of seminiferous tubules (Fig. 4 C). Percentage of cells with damaged DNA increased after vitrification of tissues from both adult and juvenile cats (Fig. 4D). After 24 h in vitro culture, warmed tissues from adult cats presented higher percentage of DNA fragmentation compared to juvenile (Fig. 4D).

Vitrification led to differential expression of 49 genes independent of age (adjusted p-value < 0.05), with 46 downregulated (7 lncRNA, 40 protein coding, 2 pseudogenes) and 3 upregulated (all protein coding) in vitrified vs. fresh tissue (Additional file 6). Hierarchical clustering of samples based on these DEGs separated adult vitrified samples from all the rest representing the biggest vitrification effect in adult tissues (Fig. 4E). One vitrified late juvenile sample (JV1) clustered with vitrified adult, indicating a bigger vitrification effect in that sample compared to the rest juvenile (Fig. 4E). Vitrified and fresh tissues from early juvenile sample(JV5, JF5) clustered together, indicating the smallest vitrification effect in that sample compared to the rest juvenile (Fig. 4E). Clustering of JF5 together with other vitrified tissues may indicate that the transcriptome of this sample is more similar to vitrified rather than fresh tissues (Fig. 4E).

Analysis of vitrification effect separately in adult and juvenile samples identified 21 DEGs in adult and no DEGs in juvenile vitrified vs. fresh tissue (adjusted p-value < 0.05; Additional file 6). To compare the effect of vitrification between adult and juvenile samples, genes satisfying condition of p-value < 0.05 and absolute fold change > 1.5 were selected for functional analysis as genes responsive to vitrification. Out of these selected genes, 18 were shared between the age groups and included 17 downregulated and 1 upregulated gene (Table 1). Out of 116 selected protein coding genes, 110 were annotated in DAVID database for domestic cat; Gene Ontology, KEGG Pathways, InterPro Domains, UniProt Keywords and SMART domains databases were used for gene set enrichment. Figure 4 F visualizes DAVID results in an Enrichment Map network. Figure 4G visualizes networks of predicted protein-protein interactions built using 109 out of 116 selected protein coding genes annotated in STRING database for domestic cat; functional enrichment was performed for each interaction cluster using STRING app, which allowed us to consider pairwise relationships among interacting genes when checking for biological significance. Additional file 7 contains the web session of both networks with interactive view and data table. Functional terms related to transmembrane were enriched in fresh testicular tissue (downregulated in vitrified vs. fresh) for both adult and juvenile ages (Fig. 4 F) and some of the DEGs from that enrichment gene set formed a protein-protein interaction cluster (Fig. 4G). Terms enriched in vitrified tissues (upregulated in vitrified vs. fresh) were related to transcription activity and TNF signaling for adult samples and to nucleus and chromosome in juvenile samples, with shared term of DNA binding enriched for both ages (Fig. 4 F). The biggest protein-protein interaction cluster included both upregulated and downregulated DEGs in vitrified vs. fresh tissue from adult and juvenile samples (Fig. 4G). Genes with the most predicted protein-protein interactions were FOS, JUNB and CREB1 (8 interactions each), JUN and EGR1 (6 interactions each), and FOSB and NFAT5 (5 interactions each).

**Table 1:** Genes responsive to vitrification in both adult and juvenile testicular tissues

Gene	Type	Fold Change		P-value		Expression	Enriched clusters/terms, Fig. 4G
		**Adult**	**Juvenile**	**Adult**	**Juvenile**		
FOSB	protein coding	2.72	2.72	0.0019	0.0198	UP	SGTR, DNA binding
TMEM41B	protein coding	-1.94	-1.67	6.65E-05	0.0326	DOWN	TM
SLC35A3		-1.56	-1.64	0.0008	0.0170		TM
SEMA6A		-1.61	-1.61	7.53E-05	0.0304		TM
DPY19L4		-1.60	-1.52	0.0362	0.0022		TM
CLCN5		-1.84	-1.55	1.69E-05	0.0083		TM, Transport
ILDR2		-2.44	-2.28	0.0008	0.0206		TM, IG
NFAT5		-1.76	-1.61	4.01E-06	0.0006		IG, Cytoplasm
DUSP16		-1.56	-1.55	0.0325	0.0427		Cytoplasm
ZNF483	protein coding	-1.66	-1.73	5.67E-05	0.0116	DOWN	No enrichment
MCTS1		-1.64	-1.62	0.0468	0.0027		
WNK3		-1.64	-1.66	7.83E-06	0.0175		
LOC101090710		-1.62	-1.68	0.0007	0.0114		
LOC102899228	lncRNA	-1.61	-1.66	0.0020	0.0394	DOWN	N/A
LOC109497486		-1.54	-1.74	0.0002	0.0060		
LOC111559448		-1.99	-1.67	4.27E-07	0.0113		
LOC111561755		-2.06	-1.85	3.05E-06	0.0060		
LOC111558666	pseudogene	-2.86	-2.07	0.0002	0.0003	DOWN	N/A

Values are taken from differential expression analysis in vitrified vs. fresh tissue, adult, and juvenile samples

Cluster names: SGTR, TNF signaling, Transcription activity; TM, Transmembrane; IG, Immunoglobulin subtype

## Discussion

This is the first overview of transcriptome dynamics coupled with structural analysis in testicular tissue from adult and juvenile domestic cats. Transcriptomic findings enabled to further characterize juvenile period, distinguishing between early and late juvenile tissues. Changes in gene expression and functional pathways were extensive from early to late juvenile to adult development stages. Additionally, tissues from juvenile animals were more resilient to vitrification compared to adult counterparts, with early juvenile sample responding the least to vitrification and late juvenile sample response being closest to adult tissues.

### Gradual testis maturation during puberty in cat

In the domestic cat, the spermatogenic function is suggested to become mature at 8 to 10 months of age with initial activation and first signs of spermatogenesis at 5 to 6 months [8, 9]. In our study, we defined juvenile period as the development period after the infant stage but prior to full adult sexual maturity to make it comparable with the recent scRNA-seq study on testis development during human puberty [4]. In juvenile samples, various degrees of maturation and germ cell differentiation were observed, from tubules with only spermatogonia in early I juvenile stage to the presence of some spermatocytes in early II juvenile to many spermatocytes and round spermatids with occasional spermatozoa in late juvenile. The expression of cellular markers associated with more differentiated germ cells also increased from early to late juvenile period, while the expression of markers associated with undifferentiated spermatogonia was highest in early juvenile period. Functional terms and pathways related to spermatogenesis and sperm motility were enriched already in late juvenile and stayed enriched in adult samples. All of this supports the gradual progressive establishment of spermatogenesis in the domestic cat beginning in late juvenile period. This is similar to humans, where low levels of incomplete spermatogenesis are observed in portions of the testis in juveniles prior to puberty, and the appearance of first spermatozoa during juvenile period does not mark completion of spermatogenic development, but the beginning of the final stages of puberty, where gradual improvement in spermatogenic efficiency is the mechanism that leads to complete maturation [38]. It is suggested that rather than sudden activation of the testis at puberty there is a slow and progressive increase of activity from mid-childhood or even earlier [38]. This also is aligned with recent scRNA-seq studies, showing that spermatogenic cell phenotypes exist on a continuum rather than in distinct subgroups separated by large transcriptome changes [39].

### Sertoli cell maturation

The present study reports downregulation of functional terms and pathways related to immune response in late compared to early juvenile period, which also coincided with the emergence of more spermatocytes and spermatids, as well as the decrease in anti-Mullerian hormone (AMH) expression. During the Sertoli cell differentiation in puberty, neighboring Sertoli cells form tight junctions, which contribute to the blood-testis barrier that permits the establishment of a special microenvironment needed for spermatogenesis [40, 41]. This barrier, together with the expression by Sertoli cells of immunoregulatory factors that actively suppress innate, humoral and cell-mediated immune responses, makes the whole testis immune privileged [42]. Around the same time, the expression of AMH in Sertoli cells decreases, which is complemented by meiotic entry of germ cells [1]. Based on this, we hypothesize that maturation of Sertoli cells begins in late juvenile period in the domestic cat. The recent scRNA-seq study in human developing testis showed that the maturation of Sertoli cells in different tubules is asynchronous and proceeds gradually, with first mature Sertoli cells starting to emerge in samples from 11 years old onward [4].

### Long non-coding RNAs in developing testis

Testis is a tissue with the most tissue-specific genes by far [17], higher fraction of genes and more diverse mRNA (less dominance of a few highly expressed genes) [43]. It is also a tissue with the highest amount of long non-coding RNAs (lncRNAs) even when comparing to brain or liver [44, 45]. lncRNAs are a large class of noncoding RNAs more than 200 nucleotides in length. Unlike mRNAs, lncRNAs exhibit unique cellular localization patterns highly correlated with the functions they perform in the cell [46, 47]. Recent studies revealed critical roles of lncRNAs during spermatogenesis [48] and accumulation of lncRNAs during meiotic and post-meiotic stages of spermatogenesis [49, 50]. The present study reports a progressive increase in expression of lncRNAs from early to late juvenile period to adult in cat testicular tissue, which may indicate on the role of lncRNAs in domestic cat spermatogenesis as well. We could not conduct functional analysis on the set of differentially expressed lncRNAs because there currently is no comprehensive functional database for domestic cat lncRNAs and lncRNAs have low sequence conservation. However, this list will inform future studies for further analysis of lncRNA functions and their interactions with mRNA and other non-coding RNAs.

### Juvenile testes are more resilient to vitrification protocol

Our results show that vitrification led to more detrimental changes in mature adult tissues compared to immature juvenile which only progressed after 24 h culture and were expressed in increased percentage of damaged seminiferous tubules and DNA fragmentation. Mature testicular tissue from adult cats also was more responsive transcriptionally to vitrification compared to juvenile. Similarly, immature testes from domestic cat were reported to be more resilient to microwave-assisted dehydration [25] and more successful in surviving and establishing spermatogenesis in xenografts [51, 52], compared to mature testes from adult cats. Specifically, we observed the downregulation of functional terms related to focal and cell-cell adhesion in adult compared to late juvenile, as well as to the whole juvenile group. Thus, the detrimental effect of vitrification and 24 h culture on testicular tissue in adult might be related to its increased sensitivity to mechanical damage due to decreased connections between cells and cells and extracellular matrix. At the same time, one sample from late juvenile period that exhibited the highest number of meiotic and post-meiotic germ cells out of all juvenile group had a similar transcriptomic response to adult samples, which might indicate the effect of the presence of these cells on tissue sensitivity to vitrification stress.

### Initial response of testicular tissue to vitrification and warming

We identified a cluster of genes responsive to vitrification in testicular tissue of adult and juvenile cats and forming predicted protein-protein interactions and enriched in transcription and TNF signaling pathway. These genes were upregulated in response to vitrification and included early response transcription factors FOS, FOSB, JUN, JUNB, and EGR1. Interestingly, the same list of genes has been reported in studies analyzing the response of different tissues to warm ischemia [53–56]. In those studies, normal and/or tumor tissues that underwent warm ischemia due to delayed processing after surgical incision demonstrated increased expression of FOS, FOSB, JUN, EGR1 as well as upregulation in immune system pathways compared to tissues processes immediately [53–56]. We hypothesize that the damage occurring in testicular tissue during vitrification is caused by the warming step and may be similar to the response of tissues to warm ischemia. Interestingly, in one study that looked at short intervals of warm ischemia, the pattern of gene and protein expression in the tissue changed within minutes following surgical excision [57]. In our protocol the initial 5-sec warming step is followed by cryoprotectant removal at room temperature for 15 min, which might be the period when testicular tissue undergoes stress similar to warm ischemia resulting in damage.

### Study limitations and next steps

In our current study, we performed bulk RNA-seq on a whole testicular tissue to have the first look on transcriptome dynamics in the developing testis of domestic cat. The addition of cell-level transcriptome information would have been invaluable to our study, however, there has been no scRNA-seq performed so far on any of the cat tissues and, therefore, no protocols established yet. We recognize that the bulk analysis of mature RNA limits our interpretations. For example, downregulation of somatic cell markers in adult compared to juvenile samples is most likely related to the decrease in somatic to germ cell ratio (in mammals, mature testes germ cells make up to 90 % of the tubular mass, compared to less than 5–10 % in immature) [2]. Our study, however, is the first necessary step in unraveling transcriptome dynamics of the developing testis in the domestic cat and the effect of age on tissue response to preservation protocols. Future studies focusing on cell-specific expression and post-transcriptional mechanisms, as well as linking transcriptomic and proteomics [58] would be essential to understand the complex process of puberty in cats, as well as tissue resilience.

Due to unavailability of domestic cat samples from the infant stage, we could only analyze testis development starting from early juvenile period. In future, adding neonatal tissues to the analysis, as well as including more samples from juvenile period, would contribute greatly to our understanding of the full timeline of testis development in domestic cat.

## Conclusions

Our study generated a high-quality transcriptomic data for testicular tissue of adult and juvenile domestic cats, which provides an important resource for future studies on testis development, spermatogenesis and fertility in cats, as well as new insights into tissue resilience. Transcriptomic data can also contribute to identification of druggable protein targets in male reproductive tracts and development of male contraception [59]. Our study provides a network of functional terms and pathways that are affected by age in the domestic cat and are either enriched in adult or juvenile testicular tissues. The interactive view of our network allows navigation through many enriched terms, their interconnection, and the associated set of genes from our study. This is the first study providing data on transcriptomic dynamic coupled with structural analysis in the cat testis according to the age and exposure to cryopreservation. Collective findings will also enable the optimization of testicular tissue preservation.

## Materials and methods

### Collection of testicular tissues

Testes from juvenile (3 to 6 months old) and adult (over 1 year old) male domestic cats were collected on different days after routine orchiectomy at local veterinary clinics and transported in phosphate buffered saline (PBS) at 4 °C to the laboratory within 6 h of excision. Testes were washed once with PBS, dissected from surrounding tissues and cut in pieces of approximately 2–3 mm^3^ in handling medium composed of Hepes-Ham’s F10 medium (Irvine Scientific, Santa Ana, CA) supplemented with 1mM pyruvate, 2 mM L-glutamine, 100 IU/mL penicillin, 100 µg/mL streptomycin, 2.5 % fetal bovine serum (FBS). For each animal, tissue pieces were either (1) fixed overnight in Bouin’s solution (histomorphology) or 4 % paraformaldehyde in PBS (TUNEL analysis), embedded in paraffin and sectioned at a thickness of 5 μm, (2) incubated overnight in RNAlater^tm^ solution (Invitrogen, Carlsbad, CA) for RNA isolation, or (3) processed for vitrification and/or in vitro culture as described below (Fig. 1). All chemicals and reagents were purchased from Sigma-Aldrich (St. Louis, MO), unless otherwise indicated.

### Vitrification and warming

Vitrification and warming was performed using modified protocol reported previously for domestic cat testicular tissue [26]. Tissue biopsies were exposed to equilibration solution (1.4 M dimethyl sulfoxide (DMSO) + 1.4 M glycerol + 0.25 M sucrose in Ham’s F10) for 10 min at room temperature (~ 22 °C) followed by a vitrification solution (2.8 M dimethyl sulfoxide (DMSO) + 2.8 M glycerol + 0.5 M sucrose + 10 % FBS in Ham’s F10) for 5 min at room temperature (~ 22 °C), then placed in cryotubes and plunged directly into liquid nitrogen and stored for at least one week in liquid nitrogen. Warming was performed by immersing cryotubes in a water bath at 50 °C for 5 s. Tissue fragments were then transferred to a sucrose gradient (0.50 M; 0.25 M; 0.00 M in Hepes-Ham’s F10 and 20 % of FBS) for 5 min at each step at room temperature (~ 22 °C) in order to remove the cryoprotectants. Warmed tissues were fixed for histology and RNA isoltation as described above or processed for in vitro culture.

### In vitro culture

Tissue culture was performed using the same protocol reported previously for domestic cat [26]. Tissue fragments were placed into 1cm^3^ pieces of 1.5 % agarose gel that were pre-conditioned by immersion in culture medium composed of Hepes-Ham`s F10 (supplemented with 2mM L-glutamine, 1mM pyruvate, 100 IU/ml penicillin, 100 µg/ml streptomycin and 5 % FBS). Two tissue biopsies on each gel were incubated for 24 h in a 4-well culture plate with 400 µl of culture medium at 38.5 °C in a humidified atmosphere of 5 % CO_2_ in air.

### Assessment of tissue histomorphology

Testicular tissue morphology was assessed via hematoxylin-eosin staining. Samples used for RNA-seq were scored using Johnsen method for registration of spermatogenesis [35]. In short, each seminiferous tubule in testicular tissue section was given a score from 10 to 1 according to the presence or absence of the main cell types arranged in the order of maturity: presence of spermatozoa scores 10, 9 or 8; spermatids (and no further) 7 or 6; spermatocytes (and no further) 5 or 4; only spermatogonia 3, only Sertoli cells 2 and no cells 1. The mean score for each sample was calculated by multiplying the number of tubuli recorded at each score with the score and then dividing the sum of all 10 multiplications by the total number of tubuli recorded [35].

Integrity of seminiferous tubules and cells was evaluated according to criteria previously established [26]. Intact tubules with no detachment of cells from the basement membrane, no rupture of stroma, no swelling of the lamina propria and normal junctions between cells were considered as a normal structure (score 1). Score 0 was attributed to tubules with changes in any of the previous criteria. A total of 30 randomly selected seminiferous tubules for each animal in each experimental group were classified as normal structure (score 1) or damage structure (score 0) totaling 180 tubules per group. Percentage of normal seminiferous tubules was calculated relative to the total number of observed tubules.

### Assessment of DNA integrity

DNA integrity was assessed using the protocol reported previously for domestic cat testicular tissue using the In-Situ Cell Death Detection kit (Roche, Basel, Switzerland) [25]. Deparaffinized rehydrated sections were rinsed twice with 0.05 % Triton X-100 in PBS for 5 min each, permeabilized with 0.5 % Triton X-100 in PBS for 30 min, rinsed once with 0.05 % Triton X-100 in PBS for 5 min and incubated in TUNEL reaction mixture (enzyme solution with terminal deoxynucleotidyl transferase (TdT) and label solution with nucleotide polymers) for 1 h at 37 °C within a humidified darkened container. Negative control (omitted TdT) was included in each run. For positive control, tissue was incubated with recombinant DNase I for 10 min before labeling. The nucleus of all cells was stained with Hoechst 33,342 (1:100) in a humidified chamber for 10 min at room temperature and then, the slides were mounted with Vectashield mounting medium (Vector laboratories, Burlingame, CA). We evaluated 60 images per experimental group, which were captured using an Olympus BX41 epifluorescence microscope (Olympus Corporation, Tokyo Japan) with SPOT advanced software 5.0 (Diagnostic Instruments, Inc., Sterling Heights, MI). Percentage of DNA damage (TUNEL positive cells) was calculated relative to the total number of observed cells.

### RNA preparation

Twenty samples were selected for transcriptomic analysis and assigned to the following 4 groups: adult, fresh tissue (AF, *n* = 5), adult, vitrified/warmed tissue (AV, *n* = 5), juvenile, fresh tissue (JF, *n* = 5) and juvenile, vitrified/warmed tissue (JV, *n* = 5; Fig. 1). Total RNA was isolated from up to 10 mg of tissue using PureLink^™^ RNA Mini Kit with on-column DNase Set (Invitrogen); tissue was homogenized in RNA lysis buffer using TissueLyser (Qiagen, Hilden, Germany; 2 × 2 min at 30 Hz; 5 mm stainless steel beads). Concentration and purity of isolated RNA was measured with NanoDrop^™^ spectrophotometer (Thermo Fisher Scientific, Waltham, MA); RNA integrity was assessed using 2100 Bioanalyzer instrument (Agilent Technologies, Santa Clara, CA). Purified RNA was stored in nuclease-free water at -80 °C until library preparation.

### Library preparation and transcriptome sequencing

Only samples with RIN ≥ 7 were used for library preparation. Sequencing libraries were generated using TruSeq Stranded mRNA LT Sample Prep Kit from Illumina (San Diego, CA) according to the manufacturer’s recommendations. In short, the workflow included randomly fragmenting total RNA for short read sequencing, reverse transcribing fragmented RNA into cDNA, ligating adaptors onto both ends of the cDNA fragments, amplifying cDNA and selecting fragments with insert sizes between 200 and 400 bp. The libraries were sequenced 150 bp paired-end using an Illumina NovaSeq 6000 System at the Psomagen Inc. (formerly Macrogen Corp., Rockville, MD) attempting 30 million reads depth per sample and 5 biological replicates per group.

### Quality control

The quality of produced data was determined by the phred quality score at each cycle using FastQC (v. 0.11.7). Trimmomatic (v. 0.38) [60] program was used to remove adapter sequences and bases with base quality lower than three from the ends. Using sliding window method, bases of reads that did not qualify for window size 4 and mean quality 15 were trimmed. Afterwards, reads with length shorter than 36 bp were dropped to produce trimmed data. Quality information for each sample after trimming is provided in Table S2.

### Reads mapping and gene expression levels quantification

Trimmed reads were mapped to reference genome GCF_000181335.3_Felis_catus_9.0 with HISAT2 (v. 2.1.0) [61], which is known to handle spliced read mapping through Bowtie2 (v. 2.3.4.1) aligner, splice-aware aligner. Table S3 shows the statistic obtained from HISAT2. After the read mapping, known genes and transcripts were assembled with StringTie (v. 1.3.4d) [62, 63] based on reference genome model. After assembly, the abundance of gene/transcript was calculated in the read count and normalized value as FPKM (Fragment per Kilobase of transcript per Million mapped reads) for a sample.

### Differential expression analysis

Differentially expression analysis was performed on data obtained from 20 samples using the DESeq2 R package [64]. For visualization, size factors were estimated from the count data and the Relative Log Expression (RLE) normalization was used to obtain regularized log transformed values. These normalized values were then used for principal component analysis (plotPCA function in DESeq2 R package) and creation of clustered heatmaps (pheatmap R package). Wald test (age effect) and likelihood ratio test (vitrification effect) were used on genes that passed an independent filtering step and resulting P values were adjusted for multiple testing using the Benjamini-Hochberg procedure.

### Functional enrichment analysis using DAVID and Enrichment Map

Selected genes from differential expression analysis were used for gene-set functional enrichment analysis with DAVID tool [65], setting species to domestic cat. For each comparison pair, total number of genes and separately up- and downregulated genes were analyzed. EASE score (modified Fisher Exact p-value of enrichment) was set to 0.05. Functional enrichment network was built based on DAVID output charts of gene-set enrichment for each comparison pair using Enrichment Map app (v. 3.3.0) [66] in Cytoscape software (v. 3.8.0) [67, 68] with Overlap set to 0.5. Autoannotate App (v. 1.3.3) with MLC algorithm based on similarity coefficient was used to create annotated groups.

### In silico protein-protein interaction analysis using STRING

In silico protein-protein interaction analysis of selected genes was performed on the basis of the STRING database for the domestic cat [69, 70]. Interaction network was built based on the list of selected genes from each comparison pair using stringApp (v. 1.5.1, [71]) in Cytoscape with confidence cutoff score set to 0.4. Functional enrichment of formed clusters was performed using domestic can genome as a background, enriched terms were analyzed with varying redundancy cutoff settings.

### Statistical analysis

Statistical analysis of RNA-seq data is provided above. For histomorphology and TUNEL analyses, data were expressed as mean and standard error and analyzed using the statistical software graphpad prism version 5.01 (GraphPad Software Inc., San Diego, CA). Data was tested for normality (Shapiro–Wilk test) and homoscedasticity (Levene’s test). Analysis of variance (ANOVA) followed by Tukey test was used to compare the effect of vitrification and 24 h in vitro culture in different experiment groups.

## Supplementary information

Additional file 1 Differentially expressed genes in fresh testicular tissue of adult compared to juvenile cat; Wald test, adjusted p-value < 0.05, absolute fold change > 2.

Additional file 2 Differentially expressed genes in fresh testicular tissue of adult compared to late juvenile cat; Wald test, adjusted p-value < 0.05, absolute fold change > 2.

Additional file 3 Differentially expressed genes in fresh testicular tissue of late compared to early juvenile cat; Wald test, adjusted p-value < 0.05, absolute fold change > 2.

Additional file 4 Differentially expressed genes in fresh tissue shared between all comparison pairs adult vs. late juvenile, adult vs. early juvenile and late vs. early juvenile (Sheet-1) or only between comparison pairs adult vs. late juvenile and late vs. early juvenile (Sheet-2); Wald test, adjusted p-value < 0.05, absolute fold change > 2.

Additional file 5 Web session interactive view with data table of Enrichment Map networks for comparison pairs adult vs. juvenile, adult vs. late juvenile and late vs. early juvenile. For description and legends refer to Fig. 3 A (adult vs. juvenile) and Fig. 3B (adult vs. late juvenile, late vs. early juvenile).

Additional file 6 Genes affected by vitrification independent of age; likelihood ratio test, condition effect, adjusted p-value < 0.05 (Sheet-1). Genes responsive to vitrification protocol in adult (Sheet-2) and juvenile (Sheet-3) samples; likelihood ratio test, p-value < 0.05, absolute fold change > 1.5.

Additional file 7 Web session interactive view with data table for Enrichment map and STRING network for genes responsive to vitrification in adult and juvenile samples. For description and legend refer to Fig. 4 F (Enrichment map) and Fig. 4G (STRING network).

Additional file 8 **Table S1. **Johnsen score counts for testicular samples used in RNA-seq analysis.

Additional file 9 **Table S2.** Trimmed data statistics.

Additional file 10 **Table S3.** Mapped data statistics.

## Data Availability

All sequence data was generated in the current study and deposited to the NCBI SRA repository, BioProject (PRJNA662384). The differential gene expression datasets supporting the conclusions of this article were generated in the current study and are included within the article as additional files.
