# Supplementary material for: Transcriptome dynamics in developing testes of domestic cats and impact of age on tissue resilience to cryopreservation
Source: BMC Genomics. 2021 Nov 23;22:847. doi: 10.1186/s12864-021-08099-8 (PMC8611880; doi:10.1186/s12864-021-08099-8)
Supplement: Supplementary file 8 — Table S1. Johnsen score counts for testicular samples used in RNA-seq analysis. [file 12864_2021_8099_MOESM8_ESM.docx]

**Table S1. Johnsen score counts for testicular samples used in RNA-seq analysis**

| Sample ID | JF1 | JF2 | JF3 | JF4 | JF5 |  | AF6 | AF7 | AF8 | AF9 | AF10 |
| --- | --- | --- | --- | --- | --- | --- | --- | --- | --- | --- | --- |
| Age group | Juvenile | | | | |  | Adult | | | | |
| 10 | 0 | 0 | 0 | 0 | 0 |  | 12 | 0 | 0 | 5 | 2 |
| 9 | 3 | 0 | 0 | 0 | 0 |  | 102 | 68 | 36 | 14 | 29 |
| 8 | 3 | 2 | 0 | 0 | 0 |  | 38 | 52 | 43 | 16 | 32 |
| 7 | 4 | 15 | 1 | 0 | 0 |  | 33 | 49 | 99 | 54 | 66 |
| 6 | 14 | 83 | 43 | 4 | 0 |  | 7 | 12 | 13 | 8 | 12 |
| 5 | 36 | 49 | 64 | 55 | 0 |  | 14 | 3 | 4 | 4 | 0 |
| 4 | 28 | 45 | 26 | 89 | 2 |  | 0 | 0 | 0 | 1 | 2 |
| 3 | 29 | 19 | 6 | 86 | 193 |  | 0 | 0 | 0 | 0 | 0 |
| 2 | 0 | 0 | 0 | 0 | 5 |  | 0 | 0 | 0 | 0 | 0 |
| 1 | 0 | 0 | 0 | 0 | 0 |  | 0 | 0 | 0 | 0 | 0 |
| Mean Score | 4.6 | 4.9 | 4.6 | 3.7 | 3.0 |  | 8.1 | 7.9 | 7.5 | 7.3 | 7.5 |

Sample IDs represent age (A, adult; J, juvenile), tissue condition (F, fresh) and animal (1 to 10).
