# Supplementary material for: Transcriptome dynamics in developing testes of domestic cats and impact of age on tissue resilience to cryopreservation
Source: BMC Genomics. 2021 Nov 23;22:847. doi: 10.1186/s12864-021-08099-8 (PMC8611880; doi:10.1186/s12864-021-08099-8)
Supplement: Supplementary file 9 — Table S2. Trimmed data statistics. [file 12864_2021_8099_MOESM9_ESM.docx]

**Table S2. Trimmed data statistics.**

| **Sample ID** | **Total read bases** | **Total reads** | **GC(%)** | **Q20(%)** | **Q30(%)** |
| --- | --- | --- | --- | --- | --- |
| CatTes-JF1 | 7,796,079,782 | 56,626,008 | 50.47 | 98.76 | 95.57 |
| CatTes-JF2 | 5,572,858,997 | 40,415,578 | 49.38 | 98.75 | 95.57 |
| CatTes-JF3 | 4,112,513,750 | 30,114,048 | 50.29 | 98.49 | 94.89 |
| CatTes-JF4 | 4,462,681,864 | 32,414,504 | 50.05 | 98.43 | 94.73 |
| CatTes-JF5 | 5,054,677,648 | 37,063,612 | 51.19 | 98.49 | 94.92 |
| CatTes-JV1 | 4,637,757,507 | 34,054,818 | 51.73 | 98.59 | 95.17 |
| CatTes-JV2 | 5,234,034,904 | 38,564,578 | 50.58 | 98.7 | 95.49 |
| CatTes-JV3 | 4,950,256,111 | 36,027,640 | 50.61 | 98.48 | 94.83 |
| CatTes-JV4 | 4,849,457,493 | 35,633,024 | 50.81 | 98.51 | 94.94 |
| CatTes-JV5 | 4,272,153,264 | 31,064,192 | 51.08 | 98.69 | 95.45 |
| CatTes-AF6 | 5,098,449,991 | 37,055,478 | 50.29 | 98.7 | 95.47 |
| CatTes-AF7 | 4,755,293,640 | 34,303,878 | 49.47 | 98.48 | 94.86 |
| CatTes-AF8 | 5,123,683,472 | 37,057,142 | 49.96 | 98.68 | 95.37 |
| CatTes-AF9 | 5,237,816,517 | 37,869,322 | 50.07 | 98.66 | 95.34 |
| CatTes-AF10 | 5,346,040,789 | 38,566,508 | 49.9 | 98.65 | 95.31 |
| CatTes-AV6 | 4,810,459,187 | 34,736,670 | 49.85 | 98.69 | 95.39 |
| CatTes-AV7 | 6,215,825,067 | 45,957,748 | 50.9 | 98.7 | 95.44 |
| CatTes-AV8 | 5,155,397,070 | 37,383,526 | 50.32 | 98.54 | 95.02 |
| CatTes-AV9 | 4,549,761,657 | 32,716,624 | 50.78 | 98.61 | 95.14 |
| CatTes-AV10 | 5,170,847,421 | 37,576,784 | 49.79 | 98.72 | 95.48 |

Sample IDs correspond to IDs in BioProject PRJNA741252, NCBI SRA

Total read bases: Total number of read bases after trimming

Total reads: Total number of reads after trimming

GC (%): GC Content

Q20 (%): Ratio of bases that have phred quality score greater than or equal to 20

Q30 (%): Ration of bases that have phred quality score greater than or equal to 30
