# Supplementary material for: Transcriptome dynamics in developing testes of domestic cats and impact of age on tissue resilience to cryopreservation
Source: BMC Genomics. 2021 Nov 23;22:847. doi: 10.1186/s12864-021-08099-8 (PMC8611880; doi:10.1186/s12864-021-08099-8)
Supplement: Supplementary file 10 — Table S3. Mapped data statistics. [file 12864_2021_8099_MOESM10_ESM.docx]

**Table S3. Mapped data statistics.**

| **Sample ID** | **# of processed reads** | **# of mapped reads** | **% of mapped reads** | **# of unmapped reads** | **% of unmapped reads** |
| --- | --- | --- | --- | --- | --- |
| CatTes-JF1 | 56,626,008 | 55,643,768 | 98.27 | 982,240 | 1.73 |
| CatTes-JF2 | 40,415,578 | 39,594,252 | 97.97 | 821,326 | 2.03 |
| CatTes-JF3 | 30,114,048 | 29,521,375 | 98.03 | 592,673 | 1.97 |
| CatTes-JF4 | 32,414,504 | 31,759,823 | 97.98 | 654,681 | 2.02 |
| CatTes-JF5 | 37,063,612 | 36,387,506 | 98.18 | 676,106 | 1.82 |
| CatTes-JV1 | 34,054,818 | 33,439,710 | 98.19 | 615,108 | 1.81 |
| CatTes-JV2 | 38,564,578 | 37,786,373 | 97.98 | 778,205 | 2.02 |
| CatTes-JV3 | 36,027,640 | 35,341,489 | 98.1 | 686,151 | 1.9 |
| CatTes-JV4 | 35,633,024 | 34,939,156 | 98.05 | 693,868 | 1.95 |
| CatTes-JV5 | 31,064,192 | 30,453,601 | 98.03 | 610,591 | 1.97 |
| CatTes-AF6 | 37,055,478 | 36,262,580 | 97.86 | 792,898 | 2.14 |
| CatTes-AF7 | 34,303,878 | 33,552,034 | 97.81 | 751,844 | 2.19 |
| CatTes-AF8 | 37,057,142 | 36,269,408 | 97.87 | 787,734 | 2.13 |
| CatTes-AF9 | 37,869,322 | 37,093,283 | 97.95 | 776,039 | 2.05 |
| CatTes-AF10 | 38,566,508 | 37,722,512 | 97.81 | 843,996 | 2.19 |
| CatTes-AV6 | 34,736,670 | 34,018,614 | 97.93 | 718,056 | 2.07 |
| CatTes-AV7 | 45,957,748 | 45,050,286 | 98.03 | 907,462 | 1.97 |
| CatTes-AV8 | 37,383,526 | 36,612,015 | 97.94 | 771,511 | 2.06 |
| CatTes-AV9 | 32,716,624 | 32,061,094 | 98.0 | 655,530 | 2.0 |
| CatTes-AV10 | 37,576,784 | 36,810,090 | 97.96 | 766,694 | 2.04 |

Sample IDs correspond to IDs in BioProject PRJNA741252, NCBI SRA

Processed reads: Number of cleaned reads after trimming

Mapped reads: Number of reads mapped to reference

Unmapped reads: Number of reads that failed to align
